# Microwave-assisted extraction of cellulose nanocrystals from almond (*Prunus amygdalus*) shell waste

**DOI:** 10.3389/fnut.2022.1071754

**Published:** 2023-01-24

**Authors:** Arantzazu Valdés, Gurutz Mondragon, María Carmen Garrigós, Arantxa Eceiza, Alfonso Jiménez

**Affiliations:** ^1^Department of Analytical Chemistry, Nutrition and Food Science, University of Alicante, San Vicente del Raspeig, Spain; ^2^Materials Technologies Group, Chemical and Environmental Engineering Department, University of the Basque Country - UPV/EHU, Donostia-San Sebastián, Spain

**Keywords:** *Prunus amygdalus*, almond shell waste, microwave-assisted extraction, cellulose nanocrystals, experimental design, valorization

## Abstract

Almond (*Prunus amygdalus*) is one of the most common tree nuts on a worldwide basis. This nut is highly regarded in the food and cosmetic industries. However, for all these applications, almonds are used without their shell protection, which is industrially removed contributing approximately 35-75% of the total fruit weight. This residue is normally incinerated or dumped, causing several environmental problems. In this study, a novel cellulose nanocrystal (CNCs) extraction procedure from almond shell (AS) waste by using microwave-assisted extraction was developed and compared with the conventional approach. A three-factor, three-level Box–Behnken design with five central points was used to evaluate the influence of extraction temperature, irradiation time, and NaOH concentration during the alkalization stage in crystallinity index (CI) values. A similar CI value (55.9 ± 0.7%) was obtained for the MAE process, comprising only three stages, compared with the conventional optimized procedure (55.5 ± 1.0%) with five stages. As a result, a greener and more environmentally friendly CNC extraction protocol was developed with a reduction in time, solvent, and energy consumption. Fourier transform infrared (FTIR) spectra, X-ray diffractogram (XRD), atomic force microscopy (AFM), and scanning electron microscopy (SEM) images, and thermal stability studies of samples confirmed the removal of non-cellulosic components after the chemical treatments. TEM images revealed a spherical shape of CNCs with an average size of 21 ± 6 nm, showing great potential to be used in food packaging, biological, medical, and photoelectric materials. This study successfully applied MAE for the extraction of spherical-shaped CNCs from AS with several advantages compared with the conventional procedure, reducing costs for industry.

## Introduction

Almond (*Prunus amygdalus*) is one of the most common tree nuts worldwide. The global production of almond has increased significantly in the past years from 2.4 million tons in 2009 up to 4.1 million tons in 2020 (1). The USA is the top worldwide producer of almonds, with an estimated annual production of 2.4 million tons. Spain is the second largest producer with approximately 0.42 million tons, followed by Iran, Turkey, Australia, and Morocco. Almonds are highly regarded in the gastronomy and food industry due to their organoleptic properties (2–4). They can be also used as an ingredient in processed foods added to confectionary and bakery products (5, 6). Almonds are also used as oils in cosmetics and in the medical field due to their anti-inflammatory properties (7, 8). For all these applications, almonds are used without their shell or seed protection, which is industrially removed, contributing approximately 35-75% of the total fruit weight (9, 10). Consequently, approximately 0.8–1.8 million tons of almond shells (AS) are left annually.

Almond shell is the lignocellulosic material forming the husk of the almond tree fruit with no important industrial usage since it is normally incinerated or dumped. As a consequence, the value of this agricultural residue is reduced, causing several environmental problems such as air pollution, soil erosion, and decrease in biological activity in soils (11, 12). To overcome these concerns, the valorization of agro-food by-products has received growing attention in the last few years as a mean for farmers to have a second income from plantation, producing value-added compounds and contributing to a sustainable growth based on a circular economy (13, 14). To date, AS has been used in different applications such as in the preparation of activated carbon (15, 16), low-cost bioadsorbent from contaminated solutions (17, 18), and reinforcement material in different matrices such as polypropylene (19, 20) polylactic acid (21, 22), or starch-based biocomposites (23). In this context, the main chemical constituents of AS are cellulose, hemicellulose, and lignin (24, 25). In particular, cellulose nanocrystals (CNCs) are a crystalline form of cellulose existing in plant matter together with amorphous cellulose (26). It presents unique advantages and properties, such as biodegradability, biocompatibility, renewability, relatively high resistance, and recyclability (27), which can be employed in different areas such as food packaging, photoelectric materials, and biological medicine (28).

To valorize the cellulose fractions of AS, several chemical treatments have been studied. Among the most studied processes, the isolation of CNCs has been widely reported in the literature (29–33). As a first step, the removal of non-cellulosic compounds such as lignin is necessary. Thus, alkaline and bleaching chemical treatments stand out by using different alkali agents, such as NaOH, causing biomass swelling and the removal of the non-cellulosic compounds, followed by acetylation and acid hydrolysis chemical treatments. The disordered regions of cellulose are hydrolyzed, whereas crystalline regions have a higher resistance to the acid attack. In general, these chemical treatments are carried out in a wide range of temperatures (45–250°C) and are normally time-consuming procedures. As a consequence, the scientific and industry sectors are getting interested in exploring novel extraction technologies to revalorized by-products from the agricultural and food matrices. Recently, microwave-assisted extraction (MAE) procedures were developed, showing benefits that transcend conventional methods. Among them, good reproducibility is achieved and the sample is minimally manipulated, carrying out the extraction procedure with a reduction in the solvent volume, exposure time, and energy consumption (34, 35). This technique has been successfully used with effective CNC extraction in different matrices, such as seaweed (36), jute (37), apple pomace (38), cotton (39), and wood (40). Nevertheless, no MAE application for the extraction of CNCs from AS has been found in the literature.

The aim of this study was the development of a new green approach for the extraction of CNCs in AS by MAE for the first time, reducing costs in the food industry and overcoming the main disadvantages of conventional extraction procedures. MAE experimental parameters were optimized by response surface methodology (RSM) using a Box–Behnken design (BBD). The obtained MAE results were compared with those obtained from an optimized conventional procedure, and CNC samples were fully characterized using structural (FTIR, XRD), morphological (SEM, AFM, TEM), and thermal (TGA) analytical techniques.

## Materials and methods

### Materials and sample preparation

Almond shell industrial by-product from Marcona almond cultivar was obtained from “Sirvent Almendras S.A.” (Alicante, Spain). Before analysis, 100 g of AS was washed with 500 ml of cold-distilled water, followed by a drying process at ambient temperature for 12 h and a final drying process at 40°C for 4 h. A fine AS powder was obtained by two grinding steps. First, AS was ground in a domestic grinder (Fagor, Spain) for 15 s to reduce its initial size. Then, the obtained sample was pulverized to a fine powder using a high-speed rotor mill (Ultra Centrifugal Mill ZM 200, RETSCH, Haan, Germany) equipped with a 1-mm sieve size.

The reagents used in this work for the specific chemical treatments were supplied by Panreac (Barcelona, Spain): sulfuric acid (PA-ISO, 96 wt.%), toluene (HPLC grade), glacial acetic acid (QP), sodium hydroxide (PA-ACS-ISO), ethanol (PA-ACS, 96% v/v), and nitric acid (PA-ISO, 65 wt.%).

### AS chemical composition

The acid-insoluble lignin (T222 om-02), moisture content (T264 cm-97), ash content (T211 om-02), hot water solubility (T207 cm-99), sodium hydroxide solubility (T212 om-02), and ethanol–toluene extractable content (T204 cm-97) were determined based on the corresponding Technical Association of Pulp and Paper Industry standards. The holocellulose content in raw materials was determined as described by Wise et al. (41). The hemicellulose content of AS was calculated as the difference between the holocellulose and α-cellulose contents (27).

### Conventional CNC extraction

To obtain CNCs from AS, different successive specific chemical treatments were carried out in the following order (42): extractives, pre-alkalization, alkalization, acetylation, and acid hydrolysis. Table 1 shows the main chemical reaction conditions used for the optimization of the CNC conventional extraction process. The extractives removal (waxes and oils) and pre-alkalization stages were carried out according to Mondragon et al. (27). The pre-alkali chemical treatment on cellulose fiber is a swelling process in which the natural crystalline structure of the cellulose relaxes (43). Then, the sample was filtered through a glass microfiber filter and washed with distilled water until the liquid filtrate was at pH 7–8. Finally, the sample was dried in an oven at 60°C for 24 h. After extractives removal and pre-alkalization, alkalization, acetylation, and acid hydrolysis steps were carried out at different times, following the procedure described in Table 1. The final product was dialyzed in deionized water for 4 days until reaching pH 4–5 to remove any free acid molecules from the dispersion, and then the obtained suspension was finally lyophilized for further analysis.

**TABLE 1:** Chemical reaction conditions used for obtaining CNCs from AS by following the conventional procedure.

Treatment	Chemical reaction	Time	Sample code
Extractives	Ethanol/toluene (1:2, v/v); Under reflux fiber/acid ratio (1:38, w/v)	6 h	E
Pre-alkalization	2 wt.% NaOH solution; 100°C fiber/acid ratio (1:33, w/v)		PA
Alkalization	7.5 wt.% NaOH solution 100°C fiber/acid ratio (1:20, w/v)	90 min 120 min 240 min 12 h 24 h	A90 A120 A240 A12 A24
Acetylation	Acetic acid/nitric acid solution (6:1 v/v); 100 °C fiber/acid ratio (0.6:14, w/v)	30 min 60 min 90 min	Ac30 Ac60 Ac90
Acid hydrolysis	64 wt.% sulfuric acid 45°C fiber/acid ratio (1:10, w/v)	15 min 30 min 45 min	H15 H30 H45

The high content in lignin and other non-cellulosic compounds in AS suggested the alkalization step to be a crucial stage for CNC preparation. In this study, MAE was proposed to optimize the alkalization treatment using a FLEXIWAVE™ microwave oven (Milestone srl, Bergamo, Italy). The powdered AS sample, without carrying out the extractives and pre-alkalization stages, was stirred at 500 rpm. In total, 3.000 ± 0.001 g of homogenized AS powder was treated with 60 ml of the solvent in a 100 ml quartz flask that was connected to a vapor condenser. This AS–solvent ratio was found to be the most effective for this quantity of sample without the formation of AS aggregates in the quartz flask during the extraction stage. The system operated in the open-vessel extraction configuration allowing very efficient heating (34, 44).

Response surface methodology (RSM) was proposed to determine the optimal MAE conditions of the alkalization stage in order to improve the overall extraction of CNC from AS. For this purpose, a BBD, comprising 17 experimental runs, was used. Five central points were added to evaluate the experimental error. Experiments were carried out in randomized order to evaluate the effects of three factors at three levels: extraction temperature (45, 70, and 95°C), irradiation time (5, 15, and 25 min), and NaOH concentration (5, 7, and 9 wt.%). The levels of the experimental design were set according to the related bibliography and experimental limitations. The response obtained from the experimental design was evaluated in terms of the crystallinity index of samples (CI), which was determined by X-ray diffraction (Table 2). The nanocellulose crystallinity is considered the most important parameter to describe the relative amounts of crystalline phases in the cellulose (45). Regression analysis was used for fitting experimental data into the following empirical second-order polynomial model (Eq. 1):


(1) $$Y=\beta_{o}+\sum \beta_{i}\,X_{i}+\sum \beta_{i\,i}\,X_{i\,i}+\sum \sum \beta_{i\,i}\,X_{i}\,X_{j}$$

**TABLE 2:** Box–Behnken experimental design and crystallinity index (CI) results.

Test	Temperature (°C)	Irradiation time (min)	NaOH (wt.%)	CI (%)
1	70	5	9	52.8
2	70	25	9	55.0
3	70	15	7	54.6
4	70	15	7	55.4
5	70	15	7	55.0
6	45	15	9	54.0
7	70	5	5	54.2
8	95	25	7	56.5
9	95	5	7	54.2
10	70	25	5	56.9
11	45	5	7	51.1
12	70	15	7	54.0
13	45	15	5	54.5
14	70	15	7	54.5
15	95	15	9	58.4
16	45	25	7	51.3
17	95	15	5	56.4

where *Y* is the predicted response, X represents the variables of the system, i and j are design variables, β_0_ is a constant, β_*i*_ is the linear coefficient, β_*ii*_ is the quadratic coefficient, and β_*ij*_ is the interaction coefficient of variables i and j.

The obtained extracts were filtered through a glass microfiber filter (MFV3; filter lab) and washed with distilled water until the liquid filtrate was at neutral pH. Finally, the sample was dried in an oven at 60°C for 24 h and kept at room temperature for the following stages. Acetylation and acid hydrolysis were optimized in a similar way than explained for the conventional procedure. The final product was dialyzed in deionized water for 4 days to pH 4–5 in order to remove any free acid molecules from the dispersion, and then the suspension was lyophilized for further characterization.

### Characterization techniques

Samples obtained in the main chemical reactions (alkalization, acetylation, and acid hydrolysis) were submitted to characterization studies as these three stages were considered to produce the main changes in the fiber structure.

### Morphological analysis

The morphological characteristics of untreated and alkali-treated AS were observed using a JEOL JSM-840 scanning electron microscope (Peabody, MA, USA) under an acceleration voltage of 20 kV. Atomic force microscopy (AFM) was employed for the characterization of CNCs using a Multimode 8 with Nanoscope V Controller (Bruker) with an integrated silicon tip/cantilever. AFM height images were obtained operating in the tapping mode and the diameters of CNCs were calculated from the height profiles. Transmission electron microscopy (TEM) was also used, and samples were measured at an accelerating voltage of 80 kV. A nanocrystal solution (one drop at 0.1 w/v%) was deposited on the surface of a Cu grid covered with a thin carbon film. Digital image analysis (GATAN DigitalMicrograph 1.80.70 for GMS 1.8.0) was used for the determination of the particle dimension.

### X-ray diffraction

A Philips X’pert Pro automatic diffractometer was used to collect the patterns of samples. The equipment operates at 40 kV and 40 mA, in theta–theta configuration, equipped with a secondary monochromator with Cu-Kα radiation (λ = 1.5418 Å) and a PIXcel solid-state detector. A range of 2O from 6 to 50° was used for data collection. The CI of cellulose samples was calculated using the Segal method (32).

### Thermogravimetric analysis

Thermogravimetric analysis (TGA) was performed using a TGA/SDTA 851 Mettler Toledo instrument. Dynamic tests were run from 25 to 700°C at a heating rate of 10°C min^–1^. Tests were carried out under a nitrogen atmosphere (50 ml min^–1^) in order to prevent any thermo-oxidative degradation. From the TG/DTG curves, three parameters were obtained: initial degradation temperature (T_*ini*_); temperature of maximum decomposition rate (T_*max*_); and residual mass (%) at 700°C.

### Fourier transform infrared spectroscopy

A Nicolet Nexus 670 FT-IR spectrometer equipped with a Golden Gate Single Reflection Diamond ATR accessory was used. Spectra were recorded in the 4,000–400 cm^–1^ range using 40 scans for each sample and a resolution of 2 cm^–1^.

### Statistical analysis

The results obtained from the BBD were analyzed using the Statgraphics-Plus software 5.1 (Statistical Graphics, Rockville, MD, USA). All experiments were performed in triplicate, and results are expressed as mean values ± standard deviation (SD). Analysis of variance (ANOVA) was carried out using the SPSS commercial software, version 15.0 (Chicago, IL, USA). Tukey’s test at a *p*-value of ≤ 0.05 significance level was assessed to study the differences between values.

## Results and discussion

### Chemical composition

The main characteristic compounds present in the initial AS material were lignin (49.97 ± 3.57 wt.%) and α-cellulose (40.17 ± 0.68 wt.%). Similar results were obtained for Marcona and Largueta AS cultivars (32, 46). These results suggest that during the CNC extraction process, special attention needs to be paid to the alkalization step, which is the stage that allows removing the major content of the lignin component (47). The high amount of α-cellulose, a homopolymer of glucose consisting of β(1→4) bonds, suggests that AS should be considered a rich source of CNCs. Also, moisture content (6.65 ± 0.26 wt.%), ash (0.85 ± 0.08 wt.%), extractives (1.08 ± 0.08 wt.%), and hemicellulose (6.70 ± 0.97 wt.%) were obtained (32, 46, 48). Regarding AS solubility, hot water tests showed a value of 12.46 ± 1.44 wt.%, whereas 19.38 ± 1.84 wt.% was obtained in 1% NaOH (9).

### Optimization of conventional CNCs extraction procedure

#### Scanning electron microscopy (SEM)

**FIGURE 1:**
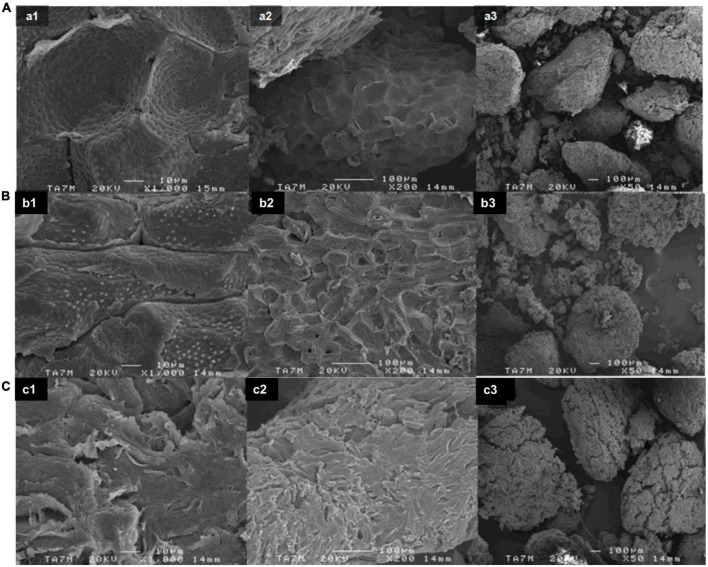
SEM images of **(A)** raw AS without any treatment, **(B)** alkalized AS for 240 min, and **(C)** alkalized AS for 24 h at (1) 1,000×, (2) 200×, and (3) 50×.

After performing the extractives and pre-alkalization stages, the alkalization stage at different times was carried out. As it has been widely reported (43), the natural fiber chemical composition consists of cellulose and other non-cellulosic compounds such as lignin, hemicellulose, pectins, and impurities such as waxes, ashes, and natural oils. Specifically, AS was shown to be rich in lignin and other minor non-cellulosic compounds such as ashes, extractives, and hemicellulose. Figure 1 shows the SEM images detailing the morphology of the studied samples after different alkali reaction times. The smooth surface of untreated AS is shown in Figure 1A. As can be observed, as the time of the alkali treatment increased (Figures 1B, C), the AS surface became rougher. This effect could indicate the partial removal of the outer non-cellulosic layer contained in AS (49). As a result, some differences in terms of surface appearance and agglomeration were observed compared with the AS raw sample.

It has been reported that the alkali treatment has two main effects on natural fibers (43). On the one hand, it increases the surface roughness as observed in Figure 1 as the alkali reaction time increased. On the other hand, it increases the amount of cellulose exposed on the fiber surface, increasing the number of possible reaction sites for the next stages of acetylation and acid hydrolysis. The type of alkali and its concentration will also influence the degree of swelling and the removal of the non-cellulosic components. Regarding sodium hydroxide (NaOH), it has been reported that Na^+^ has a favorable diameter that is able to widen the smallest pores in between the lattice planes of cellulose and penetrate them (50). Consequently, the NaOH treatment results in a higher amount of swelling. In a real application, due to the chemical constituent variation among different types of natural fiber, optimal alkalization conditions such as NaOH concentration (5–15 wt.%), time (1–48 h), and temperature (20–100°C) will vary for each sample (43). In a previous study, pectins and non-cellulosic content were fully removed from rice husks by boiling the sample in 4 wt.% NaOH solution (49). Similar results were observed when treating AS with 7.5 wt.% NaOH at different temperatures and times (30, 32). Hence, it is expected that in this study the majority of pectin and non-cellulosic content would be removed using 7.5 wt.% NaOH solution for the alkali treatment under reflux conditions for up to a maximum of 24 h.

#### X-ray diffraction (XRD) analysis

X-ray diffraction was used to investigate the crystallinity of samples after different chemical treatments. In Table 3, the CI values of samples are reported. Regarding the alkalization stage, an increase in CI was observed for the treated samples compared with raw AS, being higher after 24 h of treatment (A24). The increase in CI values could be associated with the removal of amorphous regions of lignocellulosic biomass and also of amorphous cellulose (27, 30). As a result, an optimal time of 24 h was selected for the alkali treatment to be used before the subsequent acetylation stage. These results agreed with Urruzola et al. when 24 h was also effective, causing a greater amount of delignification in AS treated with 7.5 wt.% NaOH solution (32). Regarding acetylation, an increase in CI was observed with increasing reaction time. Thus, 90 min was selected for acetylation (Ac90, Table 3) as the optimal treatment time to be used before acid hydrolysis. According to several authors (51, 52), when samples are treated with acetylation after the alkalization treatment, the amorphous region of pure cellulose is affected by the acids, and the crystallinity of all samples tends to increase. In this sense, acids such as nitric acid could cleave the inter- and intra-molecular bonds between hemicelluloses and lignin, and hydronium ions could separate cellulose from lignin and hemicelluloses (52). Finally, the hydrolysis stage performed for 15 and 30 min showed similar CI values to that obtained for Ac90, whereas 45 min drastically decreased the CI value of the samples. This fact could be explained by some sample degradation at the experimental conditions used in this work. To reduce the total time required to complete the conventional extraction procedure for CNCs preparation, 15 min was selected as the optimal time for the hydrolysis step (H15). In summary, AS should be extracted for 24 h of alkalization followed by 90 min of acetylation and 15 min of acid hydrolysis, obtaining a maximum CI of 55.5 ± 1%. These results differ from those obtained in other works found in the literature in which CI values of 72% (30) and 79% (32) were obtained for CNCs extracted from AS. This fact could be expected by the use of different experimental conditions.

**TABLE 3:** Crystallinity index (CI) values and thermal degradation parameters obtained by TGA after hydrolysis treatment at different times.

Sample	CI (%)	T_*ini*_ (°C)	T_*max*_ (°C)	Residue (%)
Raw AS	48.8 ± 0.5^a^	163 ± 2^a^	356 ± 3^a^	22 ± 1^a^
A90	50.1 ± 1.0^b^	180 ± 1^b^	321 ± 1^b^	31 ± 2^b^
A120	47.4 ± 0.8^b^	175 ± 1^c^	321 ± 2^bc^	32 ± 1^b^
A240	51.5 ± 0.2^c^	174 ± 2^c^	319 ± 1^c^	31 ± 6^b^
A12	51.6 ± 0.3^c^	186 ± 1^d^	322 ± 1^b^	31 ± 2^b^
A24	52.6 ± 0.5^d^	191 ± 1^e^	322 ± 1^b^	32 ± 1^b^
Ac30	51.4 ± 0.7^a^	154 ± 4^a^	347 ± 2^a^	25 ± 1^a^
Ac60	55.1 ± 0.5^b^	159 ± 1^a^	352 ± 3^ab^	26 ± 1^a^
Ac90	56.3 ± 0.3^c^	166 ± 2^b^	352 ± 1^b^	25 ± 1^a^
H15	55.5 ± 1.0^a^	142 ± 3^a^	293 ± 5^a^	23 ± 1^a^
H30	54.9 ± 0.9^a^	136 ± 3^a^	285 ± 1^b^	25 ± 4^a^
H45	29.2 ± 1.0^b^	124 ± 2^b^	284 ± 2^b^	22 ± 1^a^

Mean ± SD, *n* = 3. Different superscripts within the same column and chemical treatment indicate statistically significant different values (*p* < 0.05).

#### Thermogravimetric analysis (TGA)

**FIGURE 2:**
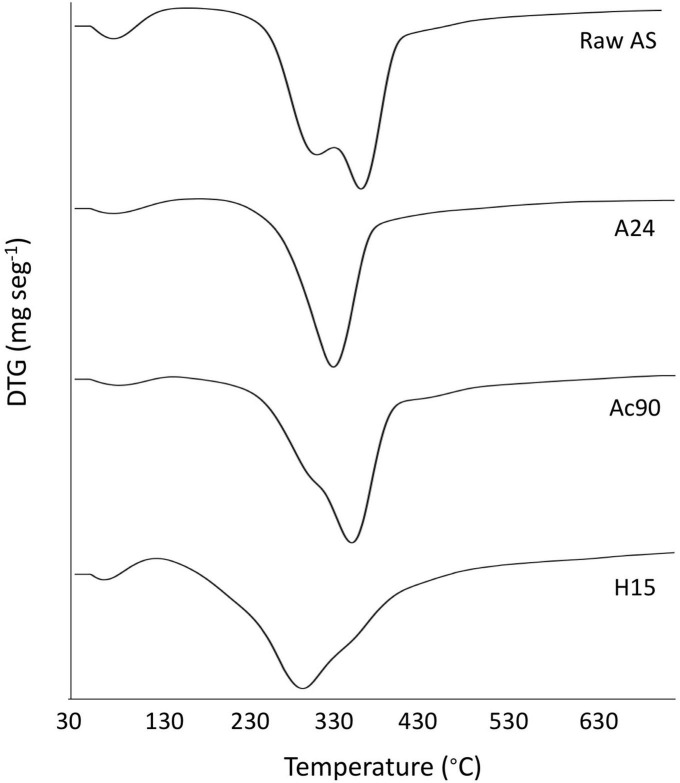
DTG curves of raw AS, A24, Ac90, and H15 samples.

The thermal stability of samples was studied by TGA. Figure 2 shows the DTG curves obtained for raw AS, A24, Ac90, and H15 samples. Four weight losses were detected for raw AS. The first one, near 100°C, corresponded to the removal of water present in the sample. The second one, near 290°C, could be probably due to the degradation of hemicelluloses and some portion of lignin (53), with one main degradation step at 356°C, corresponding to the degradation temperature of cellulose and lignin (54). The final degradation step was observed at 430°C till the end of the test corresponding to the lignin decomposition of the sample (55). As has been reported, lignin is the most difficult lignocellulosic component to be decomposed in AS and its decomposition extended to the whole temperature range, starting below 200°C and going up to 600°C (53, 56). The final residue of AS accounted for 22 ± 1% of the initial weight. These results are in accordance with those reported for AS by Urruzola et al. (32), Maaloul et al. (54), and Morales et al. (30).

After the alkalization treatment, the peak associated with the second degradation step related to the decomposition of hemicelluloses, some portion of lignin, and other non-cellulosic components disappeared (Figure 2) (27, 32). As a result, higher thermal stability was obtained for samples with an increase in T_*ini*_ values (Table 3). The highest T_*ini*_ was obtained after 24 h of treatment (A24), whereas no statistically significant differences (*p* > 0.05) were obtained between different times of alkalization for T_*max*_ and final residue values. As a result, 24 h was confirmed as the optimal studied condition of alkali treatment to be used before the acetylation stage. It is noticeable that raw AS showed a higher T_*max*_ value compared with the alkalized samples. Morales et al. (30) and Orue et al. (57) also reported similar results for alkaline-treated almond and walnut shells, respectively. These authors suggested that some prior degradation could happen during the alkaline treatment. Finally, alkalized samples showed a higher final residue content in contrast to raw AS. Some authors suggested that this difference could be attributed to inorganic impurities in the final solid (30).

Regarding acetylated samples, T_*ini*_ values decreased in contrast to the alkalized samples. This fact could be explained by considering that the acid chemical treatment allowed the initial degradation of the raw sample (58). In contrast, the acetylation step increased T_*max*_ values, being higher for the Ac90 sample. A similar behavior was reported for wheat straw and soy hulls (59), which was related to the partial removal of hemicellulose and lignin from these samples (56). In addition, the T_*ini*_ value of the Ac90 sample was the highest compared with Ac30 and Ac60 samples, with no statistical differences (*p* > 0.05) between them regarding the final residue values. Thus, the Ac90 sample, corresponding to 90 min, was confirmed as the optimal studied condition of acetylation treatment to be used before the acid hydrolysis stage. These results are consistent with the crystallinity measurements shown in Table 1.

However, as can be observed in the DTG curve of the H15 sample (Figure 2), the acid-hydrolyzed sample degradation occurred within a distinctly wider temperature range (130–530°C) than that of alkali and acetylated samples (54). As a result, after the acid hydrolysis step, the T_*max*_ values tend to decrease with increasing time (Table 3). This phenomenon was explained due to the acid-catalyzed cleavage of β-1,4-glycosidic bonds between two anhydroglucose units, and this cellulose chain reduction would result in lower thermal stability (60). Among the hydrolysis reaction times, 15 min was selected as the optimal value as these samples showed the highest thermal stability compared with those using 30 and 45 min (H30 and H45, respectively).

### FTIR spectroscopy analysis

**FIGURE 3:**
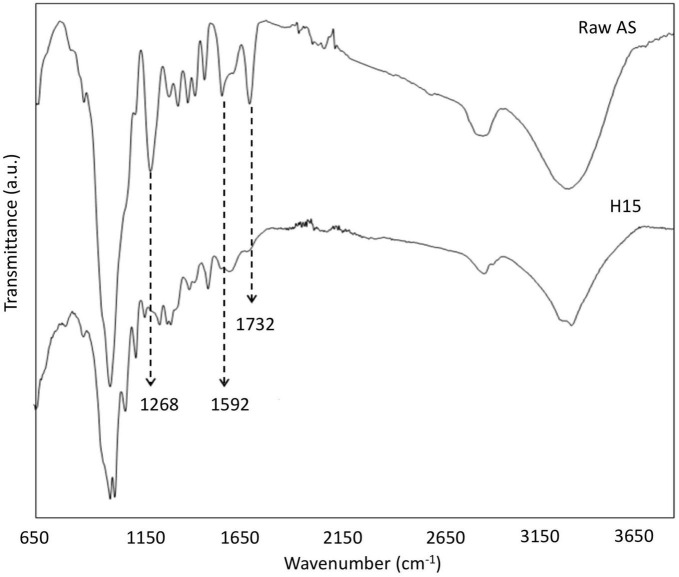
FTIR spectra of raw AS and CNCs obtained using the optimal conditions found for the conventional extraction procedure (H15).

The FTIR spectra of raw AS and CNCs obtained under the optimal conditions found for the conventional extraction procedure (H15) suggested some structural changes as the consequence of chemical treatments (Figure 3). The raw AS spectrum showed the characteristic bands of hemicellulose, cellulose, and lignin fractions of lignocellulosic materials (Table 4).

**TABLE 4:** Characteristic FTIR peaks of raw AS.

Wave number (cm^–1^)	Functional group	Fraction
3328	O–H stretching	Intramolecular hydrogen bonding from alcohols, phenols and carboxylic acids in pectin and cellulose (33)
2908 and 2846	C-H stretching	Aliphatic bonds of cellulose, lignin and hemicellulose (32, 33)
1732	C=O stretching	Carboxylic acids in lignin or the ester group in hemicelluloses (61)
1654	O–H bending	Water absorbed into the cellulose fiber structure (27)
1592	Symmetrical C=C stretching	Aromatic ring present in lignin (55)
1420	CH_2_ bending	Cellulose (62)
1226	O-H bending	Cellulose
1268	Stretching vibrations	Aromatic rings present in lignin and hemicelluloses (63, 64)
1145, 1025 and 898	C–O bridge stretching and C-H deformation vibrations	Pyranose ring skeletal of cellulose and β-glycosidic linkages between glucose units, respectively (33, 62, 65)

As shown in Figure 3, three main bands (1,732, 1,592, and 1,268 cm^–1^) tended to disappear in the CNCs spectrum of the H15 sample as a consequence of lignin, hemicellulose, and non-cellulosic compound removal. These results corroborated the successful treatment of samples under the experimental studied conditions. In addition, the appearance of the bands near 1,145, 1,025, and 898 cm^–1^ in the H15 spectrum could be related to an increase in the percentage of cellulose content as XRD and TGA results have suggested in this study.

### Atomic force microscope

**FIGURE 4:**
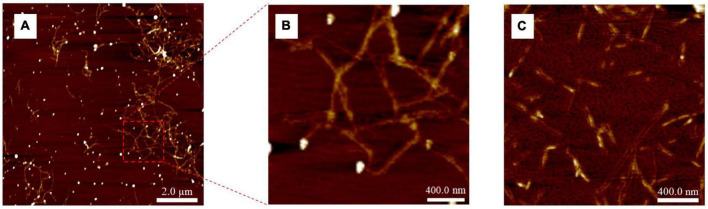
AFM images of **(A)** Ac90 (10 μm × 10 μm), **(B)** enlarged area of Ac90, and **(C)** H15 (2 μm × 2 μm).

The AFM image of the Ac90 sample (Figure 4A) confirmed the removal of hemicellulose, lignin, and the rest of the non-cellulosic materials since the defibrillation of the raw material was observed (58). After acetylation for 90 min (Ac90), some fine and large nanofibers were obtained (Figure 4B). After acid hydrolysis for 15 min (H15) (Figure 4C), a clear reduction in the length of nanofibers was observed compared with those obtained after acetylation, mainly due to the removal of the amorphous part of the cellulose AS fraction (47, 61).

The average length and diameter values obtained after the acid hydrolysis treatment were 120 ± 26 nm and 3 ± 1 nm, respectively, giving an aspect ratio of 38 ± 5, in agreement with data reported by Mondragon et al. for CNCs extracted from sisal fibers (42). According to Jiang et al. (62), the minimum aspect ratio needed for a good strength transmission for any reinforcement fiber must be kept above 10, so that it can be considered that the CNCs obtained from AS by using the optimized conventional procedure show great potential to be used as a reinforcing agent on polymer matrices.

### Optimization of CNCs MAE procedure

#### Alkalization optimization using a BBD

The almond shell was directly alkalized by MAE without performing the previous extractives and pre-alkalization steps. Table 2 shows the CI results obtained from the BBD. In this work, the adequacy of the fitted model was determined by evaluating the *F*-test obtained from ANOVA (Table 5), the lack of fit, and the coefficient of determination (*R*^2^). The CI response was expressed as a function of the independent variables using a second-order polynomial equation as follows (Eq. 2):


(2) $$\begin{array}{c} CI\left(\%\right)=68.3210-0.0096\times Temperature+0.3680\times Time\\ -5.4000\times N\,a\,O\,H-0.0003\times T\,e\,m\,p\,e\,r\,a\,t\,u\,r\,e^{2}+0.0021\\ \times T\,e\,m\,p\,e\,r\,a\,t\,u\,r\,e\times T\,i\,m\,e+0.0125\times T\,e\,m\,p\,e\,r\,a\,t\,u\,r\,e\times N\,a\,O\,H\\ -0.0126\times T\,i\,m\,e^{2}-0.0062\times T\,i\,m\,e\times N\,a\,O\,H+0.3219\\ \times N\,a\,O\,H^{2} \end{array}$$

**TABLE 5:** ANOVA results for response surface quadratic model of CNCs extraction.

Source	Sum of squares	DF	Mean square	*F*-value	*p*-value
A:Temperature	26.64	1	26.64	95.16	0.0006***
B:Time	6.84	1	6.84	24.45	0.0078**
C:NaOH	0.40	1	0.40	1.45	0.2954
AA	0.11	1	0.11	0.40	0.5628
AB	1.10	1	1.10	3.94	0.1182
AC	1.56	1	1.56	5.58	0.0775
BB	6.71	1	6.71	23.97	0.0081**
BC	0.06	1	0.06	0.22	0.6612
CC	6.98	1	6.98	24.93	0.0075**
Lack-of-fit	4.10	3	1.37	4.88	0.0799
Pure error	1.12	4	0.28		
Total (corr.)	54.96	16			

**Very significant, *p* < 0.01. ***Highly significant, *p* < 0.001.

The lack of fit test was not significant (*p*-value of 0.0799), indicating that the fitted model adequately represented the experimental data. The *R*^2^ statistic value indicated that the fitted model explained 90.50% of the variability in CI response with adjusted *R*^2^ values (88.29) quite close to *R*^2^, confirming the accuracy of the fitted model (Eq. 2) in correlating results with experimental data. In this way, the regression model represents the true relationship between the studied response and the independent variables within the range of experimental variables used (63).

According to the literature, natural fibers show different chemical compositions. Thus, it is expected that different alkalization conditions will be necessary not only for different samples, such as extraction temperature and time but also for NaOH concentration (43). In this study, extraction temperature was found to be the main significant effect on CI response (*p* < 0.05), followed by the quadratic interaction of NaOH concentration, extraction time, and, finally, the quadratic interaction of extraction time (Table 5). Regarding the effect of extraction temperature, the power of the microwave increases the temperature, causing the quick disruption of fiber cells (34). In contrast, pressure rises inside the sample vessel during MAE improving the porosity of the matrix with better penetration of the extracting solvent through the cell walls (64), increasing the release of non-cellulosic compounds to the solvent and their removal from AS. For these reasons, the positive effect of the extraction temperature on the studied response was expected. The quadratic interaction of NaOH concentration showed a significant positive effect that was related to the high lignin and other non-cellulosic materials contents in AS. Then, it was expected that as NaOH concentration increased the alkalization treatment was more efficient. The effect of the NaOH concentration from 2 to 10 wt.% was previously studied for coir fiber, where the highest studied concentration of NaOH solution provided more Na^+^ and OH^–^ ions to react with the fiber, causing a higher amount of lignin, pectin, and non-cellulosic compounds to leach out (65). Regarding extraction time, the quadratic interaction showed a negative effect in this study as using long times in samples under microwave irradiation could lead to the degradation of the cellulose chain molecules. This fact could be explained considering that cellulose has a crystalline structure due to the presence of hydrogen bonding interactions and Van der Waals forces between molecules (36). Thus, it should be expected that microwave irradiation could have a severe impact on the cellulose structure at long times due to some thermal degradation.

The optimal MAE conditions to maximize CI were determined at 95°C for 20 min using 9 wt.% of NaOH, obtaining a desirability index of 100% for the studied response. To ensure the reliability of the proposed model, verification experiments under the optimal conditions were performed, in triplicate, obtaining an experimental CI value of 69.1 ± 0.3%, which was a bit higher than the predicted value (58.5%). In conclusion, the developed quadratic model (Eq. 2) was considered reliable to optimize the CI response during the alkalization stage by MAE to obtain CNCs from AS. As a result, a higher CI value was obtained after alkalization by MAE compared with the conventional optimized procedure (52.6 ± 0.5%), reducing reaction time, energy, and solvent consumption. In addition, to the best of our knowledge, no optimization of the alkalization step by the MAE process to isolate CNCs from AS had been previously reported in the literature, highlighting the novelty of this study.

#### XRD analysis on MAE CNCs

**FIGURE 5:**
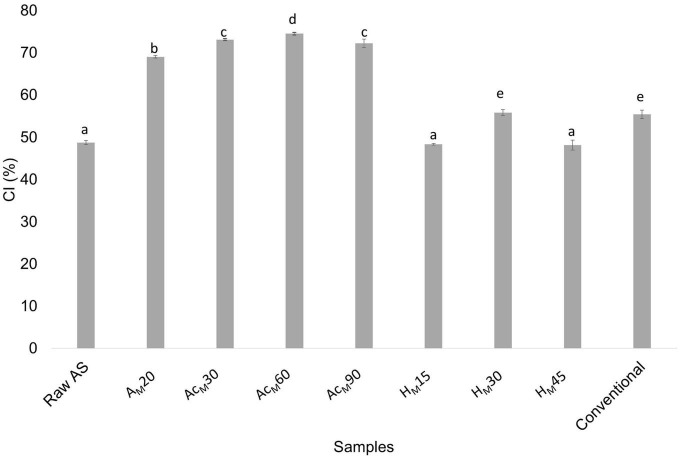
CI values obtained for raw AS; alkalized sample by MAE for 20 min (A_*M*_20); acetylated A_*M*_20 sample for 30, 60, and 90 min (Ac_*M*_30, Ac_*M*_60, and Ac_*M*_90, respectively); hydrolyzed Ac_*M*_60 sample for 15, 30, and 45 min (H_*M*_15, H_*M*_30, and H_*M*_45, respectively); and CNCs obtained using the conventional procedure (conventional). Mean ± SD, *n* = 3. Different superscripts indicate statistically significant different values (*p* < 0.05).

Figure 5 shows the CI values obtained for all stages in the MAE-based procedure compared with raw AS and CNCs obtained by the conventional procedure.

**FIGURE 6:**
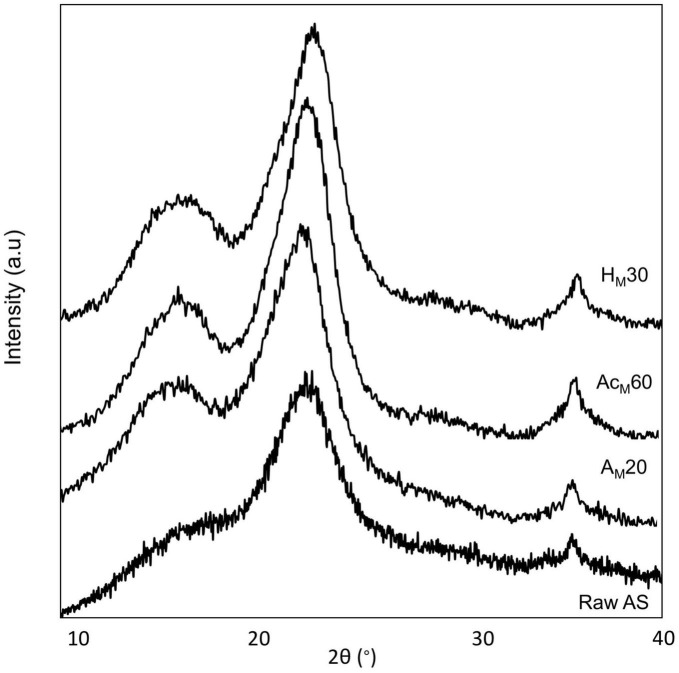
XRD patterns of raw AS, A_*M*_20, AC_*M*_60, and H_*M*_30 samples.

Regarding the acetylation step performed after the optimized alkalization by MAE, an increase in CI values was observed for all samples, being higher after 60 min of treatment (74.6 ± 0.3%). As a result, 60 min was selected as the optimal time for the acetylation treatment before the acid hydrolysis step. Regarding all studied acid hydrolysis times, it was found that the highest CI values (55.9 ± 0.7%) were obtained after 30 min, and this time was selected as the optimum for the acid hydrolysis step. As a result of this novel CNC extraction protocol, a similar value was obtained compared with the value found in the conventional procedure (55.5 ± 1.0%). However, it should be considered that the MAE-based process consisted of only three stages (alkalization for 20 min by MAE followed by 60 min of acetylation and 30 min of acid hydrolysis), being more efficient and showing a considerable reduction in time, solvent, and energy consumption. Figure 6 shows the XRD patterns of untreated AS and those obtained at different stages of the chemical treatments using the MAE procedure, followed by acetylation and acid hydrolysis. The XRD results as well as the obtained crystallinity values suggested that the cellulose crystalline structure significantly changed during the different chemical treatments used. In this sense, raw AS showed the typical pattern of semicrystalline materials with an amorphous broad hump and crystalline peaks. But as chemical treatments were carried out the samples showed three peaks typical of cellulose I around 2θ = 16°, 23°, and 35°, which were assigned to 110, 200, and 004 planes, respectively (66). These peaks became more defined upon chemical stages due to the removal of non-cellulosic compounds by the chemical treatments. Similar behavior was reported for peanut shell (67), AS (32), and sisal, hemp, and flax fibers (27).

#### Thermogravimetric analysis (TGA)

**FIGURE 7:**
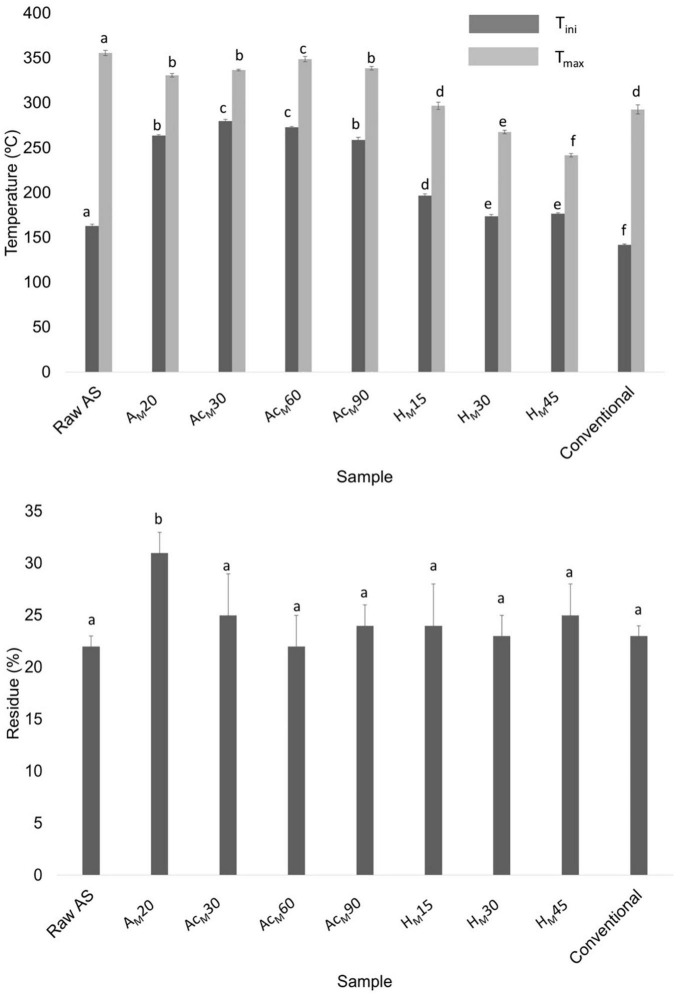
Thermal parameters obtained for raw AS; alkalized sample by MAE for 20 min (A_*M*_20); acetylated A_*M*_20 sample for 30, 60, and 90 min (Ac_*M*_30, Ac_*M*_60, and Ac_*M*_90, respectively), acid-hydrolyzed A_*M*_20 sample for 15, 30, and 45 min (H_*M*_15, H_*M*_30, and H_*M*_45, respectively); and CNCs obtained using the conventional procedure (conventional). Mean ± SD, *n* = 3. Different superscripts within the same parameter indicate statistically significant different values (*p* < 0.05).

**FIGURE 8:**
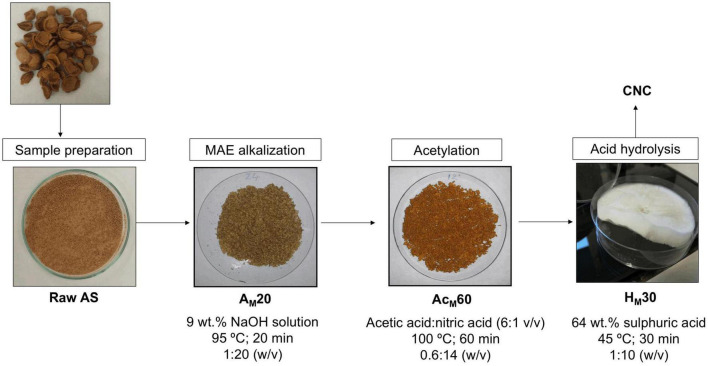
Scheme of CNCs’ isolation from AS using the MAE-based procedure.

Figure 7 shows the thermal parameters obtained in all stages of the MAE procedure compared with raw AS and CNCs obtained using the conventional procedure. Among the treated samples, the acetylated samples at 30 and 60 min showed the highest T_*ini*_ values (275 ± 2 and 273 ± 1°C, respectively). However, the Ac_*M*_60 sample showed the highest T_*max*_ value (349 ± 3°C), being the optimal selected conditions. For the acid hydrolysis, the use of 30 min showed a T_*ini*_ value of 174 ± 2°C and a T_*max*_ of 268 ± 2°C, being that the T_*ini*_ value was higher than that obtained using the conventional procedure (142 ± 3°C). Finally, no statistically significant differences (*p* > 0.05) were obtained regarding the final residue content of samples as it was previously observed when using the conventional procedure. Thus, XRD and TGA results suggested that the optimal conditions obtained in the MAE-based procedure were alkalization by MAE for 20 min using 9 wt.% of NaOH at 95°C, followed by acetylation for 60 min and acid hydrolysis for 30 min. Figure 8 shows a scheme describing this procedure to extract CNCs from AS using the MAE protocol as well as digital images of the samples obtained after each stage.

### FTIR spectroscopy analysis

**FIGURE 9:**
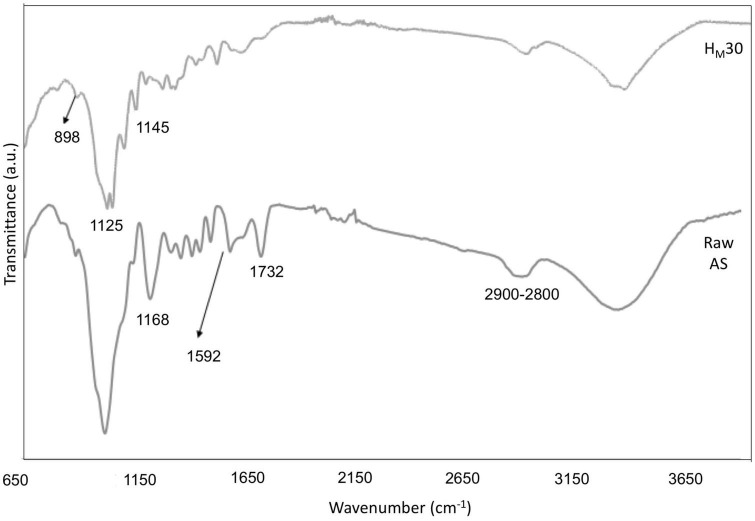
FTIR spectra of raw AS and CNCs obtained under optimal conditions of the MAE procedure (H_*M*_30).

The ATR-FTIR spectra of raw AS and H_*M*_30 sample, obtained after alkalization for 20 min by MAE followed by acetylation for 60 min, revealed similar compositional changes in the structure of samples during the chemical treatments than those obtained using the conventional protocol (Figure 9). The spectrum obtained for the untreated AS showed the characteristic bands of the amorphous fraction of the sample such as lignin with bands located in the regions close to 2,900–2,800, 1,732, 1,592, and 1,168 cm^–1^ (61, 66, 68). However, the spectrum of the H_*M*_30 sample showed the reduction or absence of these bands with an increase in the intensity of the characteristic bands of cellulose; specifically, for the bands close to 1,145 cm^–1^ (C–O–C, asymmetric stretching of cellulose), 1,125 and 898 cm^–1^ (vibration of the pyranose ring skeleton C–O–C in the cellulose fiber) (69).

### Transmission electron microscopy (TEM)

**FIGURE 10:**
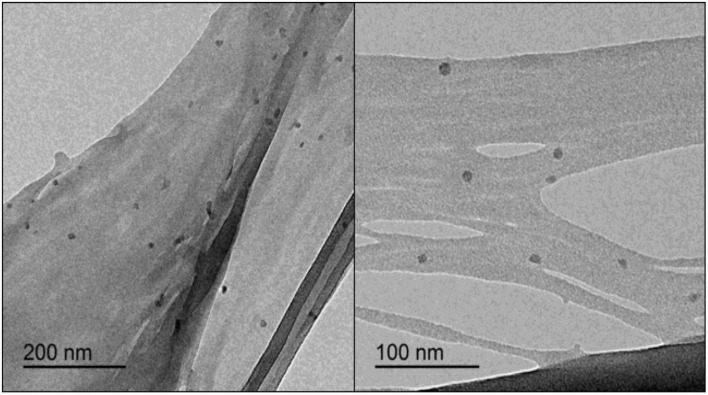
TEM images showing CNCs with spherical shape obtained under optimal conditions of the MAE procedure.

The CNCs obtained by using the MAE protocol showed a spherical shape according to TEM images (Figure 10). The average size of the obtained CNCs was 21 ± 6 nm. These results are in agreement with those reported for spherical CNCs obtained from different agricultural wastes such as sago seed shells (70) and three palm wastes (fronds, leaves, and coir) (69) having a size range of 10–15 nm and 42–81 nm, respectively.

It should be noticed that different morphologies were obtained in the present study when conventional and MAE procedures were performed. It has been widely reported that the process used for CNC preparation can affect their properties such as size and shape, as well as their potential applications (51). MAE process simultaneously produces dipolar rotation and ionic conduction (12). In dipolar rotation, the applied electric field during MAE aligned the dipoles in the matrix sample increasing the temperature and heating it as a consequence of the frictional force between molecules and the extraction medium. In ionic conduction, the dissolved charged particle oscillates forward and backward due to the applied wave. The kinetic energy of the larger ions dissipates as heat as they change direction due to friction at the molecular level. These effects generate high temperature and a pressure gradient due to confined superheating in both mechanisms (71, 72), which lead to enhanced breakup and swelling of the biomass material, increasing the contact surface area between phases (34). It was reported that the spherical CNCs structure is created by the self-assembly process of shorter cellulose fragments and rods induced by their strong interfacial hydrogen bonds (73). Thus, it is believed that shorter cellulose fragments could be formed after acetylation and acid hydrolysis treatments as a result of the prior MAE alkalization step, which allows obtaining CNCs with spherical shapes.

## Conclusion

In this study, CNCs were successfully isolated from AS, as a natural source, using MAE as an alternative energy source for alkalization treatment. An optimized conventional methodology was also used and compared with MAE. Thirty-five hours were necessary to complete the conventional CNC extraction protocol: removing the extractives for 8 h, followed by a pre-alkalization of 90 min, alkalization of 24 h, acetylation for 90 min, and finally acid hydrolysis for 15 min. The high contents of lignin and other non-cellulosic compounds present in AS suggested the alkalization step as a crucial stage of the CNCs extraction. Thus, the traditional alkalization procedure was replaced by a greener and more efficient and environmentally friendly MAE process. A similar CI value (55.9 ± 0.7%) was obtained for the MAE-assisted protocol compared with that found in the conventional procedure (55.5 ± 1.0%), consisting of only three stages (alkalization for 20 min by MAE followed by 60 min of acetylation and 30 min of acid hydrolysis) in contrast to five stages in the conventional protocol. Hence, a considerable reduction in process time from 35 h to 110 min was achieved. In addition, solvent consumption was reduced without using ethanol/toluene mixtures as the extractives step was removed from the process. Finally, the total energy consumption of the novel CNCs extraction procedure by MAE was also reduced. In this sense, the energy consumption has been estimated to be 28.6 kWh for the conventional procedure in contrast to 0.12 kWh by MAE. As a result, this study successfully applied MAE for the extraction of spherical-shaped CNCs from AS with several advantages compared with the conventional procedure, reducing the costs for the industry. The obtained CNCs show great potential to be used as a reinforcing agent in polymer composite applications such as food packaging, biological medicine, and photoelectric materials.

## Data availability statement

The raw data supporting the conclusions of this article will be made available by the authors, without undue reservation.

## Author contributions

AV: conceptualization, designed and conducted the experiments, data curation, writing—original draft and editing, methodology, and analysis and fund acquisition. GM: methodology and writing—editing. MG: supervision and fund acquisition. AE: supervision and fund acquisition. AJ: supervision and fund acquisition. All authors approved the submitted version.
